# Development of a history-taking form for mesothelioma patients at risk of exposure to asbestos

**DOI:** 10.5588/pha.25.0024

**Published:** 2025-12-03

**Authors:** R. Singh, R. Kumar, M. Nagar, S.B. Kaipilyawar, H.A. Anderson, A.V. Jadhav, T. Patel, T.R. Drischoll, E. Prriya, S.R. Yadav, S. Bishnoi, N. Singh, K. Kaur, A. Jakhetiya, N. van Zandwijk, S. Kumari, D. Khosla, S. Rajan, A.K. Verma, A. Bhatt, A. Sharma, D.K. Sinha, A. Kapoor, R. Gill, R. Dada, J. Kishore, A.L. Frank

**Affiliations:** 1 Department of Environmental and Occupational Health, Dornsife School of Public Health, Drexel University, Philadelphia, PA, USA;; 2 Department of Pulmonary Medicine, Critical Care Medicine and Sleep Disorders, Vardhman Mahavir Medical College & Safdarjung Hospital, New Delhi, India;; 3 Department of Medical Oncology, Vardhman Mahavir Medical College & Safdarjung Hospital, New Delhi, India;; 4 Public Health, Society for Health Allied Research & Education, Hyderabad, India;; 5 Department of Population Health Sciences, School of Medicine and Public Health, University of Wisconsin, Madison, USA;; 6 Department of Epidemiology, ICMR-National Institute of Virology, Pune, India;; 7 Department of Pathology, All India Institute of Medical Sciences, Rajkot, India;; 8 Sydney School of Public Health, The University of Sydney, Sydney, Australia;; 9 Thoracic Surgery, Navi Mumbai, India;; 10 Department of Pulmonary Medicine, Vallabhbhai Patel Chest Institute, University of Delhi, New Delhi, India;; 11 Thoracic Surgery, Medanta Hospital, Gurugram, India;; 12 Department of Pulmonary Medicine, Post Graduate Institute of Medical Education and Research, Chandigarh, India;; 13 Department of Pathology, Jawaharlal Institute of Postgraduate Medical Education & Research, Puducherry, India;; 14 Department of Surgical Oncology, Geetanjali Medical College, Udaipur, India;; 15 Department of Cell and Molecular Therapies, Royal Prince Alfred Hospital, Sydney Local District & University of Sydney, Sydney, Australia;; 16 Gynae-Surgical Oncology, Max Hospital, New Delhi, India;; 17 Department of Radiotherapy and Oncology, Post Graduate Institute of Medical Education and Research, Chandigarh, India;; 18 Department of Surgical Oncology, King George’s Medical University, Lucknow, India;; 19 Department of Respiratory Medicine, King George’s Medical University, Lucknow, India;; 20 Department of Surgical Oncology, Shalby Cancer and Research Institute, Ahmedabad, India;; 21 Department of Medical Oncology, National Cancer Institute, Jhajjar, (All India Institute of Medical Sciences, New Delhi), India;; 22 Department of Radiation Oncology, Indira Gandhi Institute of Medical Sciences, Patna, India;; 23 Department of Medical Oncology, Mahamana Pandit Madan Mohan Malaviya Cancer Centre & Homi Bhabha Cancer Hospital, Tata Memorial Centre, Varanasi, India;; 24 Department of Radiology, Columbia University, New York, USA;; 25 Laboratory for Molecular Reproduction and Genetics, Department of Anatomy, All India Institute of Medical Sciences, New Delhi, India;; 26 Department of Community Medicine, Vardhman Mahavir Medical College & Safdarjung Hospital, New Delhi, India.

**Keywords:** pleural mesothelioma, peritoneal mesothelioma, asbestos exposure, marble, stone-cutting, occupational health, differential diagnosis

## Abstract

**BACKGROUND:**

Mesothelioma is causally established to be primarily related to asbestos exposure as a risk factor. Before considering other possible causative factors for mesothelioma, a detailed exposure history is warranted, which looks at possible prior exposures to asbestos. Some exposures may be unknown to patients, may be forgotten or may not be readily available as a comprehensive tool for the clinicians dealing with mesothelioma patients.

**METHODS:**

We used the Delphi anonymous consensus technique to develop and validate a detailed, seven-sectioned, and comprehensive questionnaire that can be readily used by clinicians and researchers. Over two rounds, the experts followed a thorough and rigorous process to validate the questionnaire, including medical history along with occupational, non-occupational as well as para-occupational exposure to asbestos, and many other parameters that can be both rule in and rule out the possibility of mesothelioma. Questions have also been included to factor in other types of dust exposure for an informed differential diagnosis.

**RESULTS:**

We have created a readily available history-taking questionnaire validated by experts, which can be replicated for other conditions and become an essential tool for diagnosis.

**CONCLUSION:**

Apart from accurate and informed diagnosis, this documentation can be valuable for epidemiological, research, policy-informing, legal and compensation related issues.

Mesothelioma is a rare malignancy that is ‘uniquely elicited by asbestos’ and may even be a surrogate marker for asbestos exposure.^1^ Other rare sources of exposure that are causative factors for development of mesothelioma include carbon nanotubes and erionite.^2,3^ In this study, we did not attempt to list all possible exposures or conditions that can lead to development of mesothelioma, but the fact that asbestos is the key causative factor needs to expand beyond the most obviously linked exposures.^4^ Other types of asbestos exposure may come from unknown sources, or be distant exposures that are long-forgotten.^5^ Mesothelioma also has a long latency, which can hinder the ability to identify prior exposures.^6^ We therefore attempted to create a model exposure history form for clinicians dealing with potential mesothelioma cases for the differential diagnosis in case of suspected patients or for conclusive diagnosis in pathologically and otherwise clinically diagnosed patients. This may also be useful for screening, research, epidemiological, legal or compensation related issues.^6^

An illustrative example of the difficulty involved is a person who may have never had any exposure in an asbestos factory, worked or lived near a mine or disposal site of asbestos, but may have used talcum powder (with asbestos contamination naturally present) for a part of their life. Another example is a person who has not had any occupational or non-occupational exposure to asbestos, but worked as a jeweller, a naval officer, or a teacher with unknowing exposure to asbestos in the work environment. Clinicians may be unaware of such non-occupational exposures, or other sources, and patients may be unable to recall possible sources in the limited time available for consultation. It is also worth noting that there may be a delay in mesothelioma diagnosis while the most well-known exposure to asbestos are investigated, but other indirect exposures may not be considered.^7^ This may have led to some studies not attributing mesothelioma to asbestos exposure. The issue of asbestos contaminating other minerals (e.g., talcum powder, marble, or allied minerals in a coal mine) may be important but may not be readily identified by patients, caregivers or medical professionals.^8–10^

Mesothelioma is prone to misdiagnosis in medical facilities where the necessary histopathological investigations and ultra-structural studies are not available and differential diagnosis is therefore challenging.^11^ For such patients, history taking may be an important tool.^12^ Such an exercise, if done at a primary or secondary care centre, may provide for informed input for the specialists at tertiary care centres. In many countries, tertiary care centres may have a large patient load and lack of knowledge of a patient’s local language (as patients may travel from their regions to tertiary centres in metropolitan cities).^13^ The physician at the local primary or secondary level may be better at securing the patient’s history which will be useful at the tertiary level.

## METHOD

A questionnaire was developed by taking the most likely causes of asbestos exposure along with other basic clinical parameters. This questionnaire was based on literature from which the most common sources of occupational, para-occupational, or non-occupational exposure to asbestos was collated. To this was added much less common exposures known to experts with whom this was shared using the Delphi Technique for its validation.^14^ The process took place through two rounds of anonymous feedback between a moderator and 30 experts. The experts were selected from a wide range of specialities related to mesothelioma, which includes physicians dealing clinically with patients and others diagnosing the patients.

### Expert selection

30 experts were included in the validation of the questionnaire. Out of the 30, 20 dealt with mesothelioma patients clinically while the remaining 10 dealt with mesothelioma patients for diagnosis and exposure history, or were researchers involved in an allied area related to mesothelioma, or asbestos. Some, who did not deal with patients clinically were from the field of community medicine, nursing, genetics, toxicology, epidemiology, and public health. Others were pathologists who play an important role in confirmatory diagnosis of mesothelioma. The experts dealing with mesothelioma patients clinically included medical oncologists, surgical oncologists, pulmonologists, radiation oncologists, internal medicine physicians and occupational physicians.

### Questionnaire for physicians for validation

The final questionnaire for validation is presented in the Supplementary Data. The questionnaire comprises 7 sections with one section dealing with patient background, occupation, geographical region of work and residence. The other 6 sections were arranged from ‘A’ to ‘F’. Section A deals with patient medical history. This is especially related to TB as some, if not many, patients who have mesothelioma, may have had asbestosis first, and were in the past incorrectly diagnosed with TB and administered anti-TB medicines. There are other questions related to ruling out types of pulmonary diseases (whether restrictive or obstructive). There are additional questions related to diagnosis and treatments which may be useful for patients who may not know the details of their condition but may know about past medical care received. This is particularly useful as many workers may not be aware of medical terminology, and questions, for example about whether a CT scan has ever been done may serve as a useful clue for the kind of past medical examination the patents would have undergone. Section B deals with a detailed history of the occupation of the patient which not only includes questions to rule in asbestos exposure but also questions which may possibly rule out asbestos exposure. It provides a detailed list of possible occupations shown in the literature to lead to asbestos exposure and in some cases attributable to occurrence of mesothelioma. Some sources with citations are listed in discussion section below. Section C primarily deals with environmental exposures, where a person may be exposed to asbestos in settings including living and working near asbestos sources (e.g., mines, factories, construction sites, etc). Section D mainly deals with non-occupational sources of asbestos exposure, where the issues of using asbestos containing products is covered. Section E deals with the para-occupational sources of asbestos exposure. This relates to Section B, but is for relatives and people within the same household of the patient who may have been exposed to asbestos at work and be a source of exposure through proximity. Section F deals with other exposures, which may be useful in defining asbestos exposure and identifying the possibility of other exposure which may be useful in differential diagnosis of other conditions. The questionnaire was based on a narrative review of the literature (with some sources described in the Supplementary Data), and others were contributed by the experts by their clinical experience.

### Validation of the questionnaire

In the first round, the 7 sections were separately presented to all the experts in the form of an online form where the experts either accepted a question/section as it is, rejected it as a whole or provided feedback. The individual responses of experts were collected integrated into a revised questionnaire which was sent again in Round 2 to the experts to provide feedback. Anonymity was maintained with no expert having knowledge about the response of the other experts.

## RESULTS

The result of the consensus after two rounds led to the creation of the detailed questionnaire for history-taking of mesothelioma patients by clinicians which is present in the Supplementary Data. The details of the two rounds of the Delphi consensus building are present in the Table. Issues commented upon by the experts include the issue of misdiagnosis of patients as many mesothelioma patients are on anti-TB drugs or have taken these in the past when they approach a clinic, where their mesothelioma was diagnosed. Other medical history parameters have been included to rule out, rather than to rule in mesothelioma. This history questionnaire will serve as a useful tool for primary and secondary levels to guide patients to the correct treatment at the tertiary level, and the tool will strengthen history-taking and allow clinicians to understand the occupational, environmental, and non-occupational exposure histories of patients. This will allow for a quicker and more accurate diagnosis, and follows the suggestion of the father of occupational medicine, Bernardino Ramazzini, who suggested that physicians should add to the list of questions for their patients: ‘What occupation does he follow?’.

**TABLE.** Details of the two rounds of the Delphi consensus for the validation of the history-taking questionnaire for mesothelioma patients by clinicians.

Description of Section	Round 1: Sharing Online, Section wise questionnaire for validation.	Round 2: Sharing the revised complete questionnaire for validation.
Section dealing with occupation status.	22 experts accepted section as it is (∼73%). 8 experts suggested revisions (∼27%). None rejected. These mostly pertained to a more detailed and nested description to be added.	8 experts suggested further changes to the revised version which had revisions integrated from Round 1.
Section A: Deals with patient medical history	16 experts accepted section as it is (∼53%). 14 experts suggested revisions (∼47%). None rejected. This included many revisions including taking detailed history-smoking, cough, cancer in family, etc.	Comments included: Detailing out smoking and cough history, adding some more exposure scenarios; detailing out weight loss and making the occupation history more detailed.
Section B: Deals with a detailed history of the profession of the patient.	20 experts accepted section as it is (∼67%). 10 suggested revisions (∼33%). None rejected. This included many more possible occupations from around the world to make it more comprehensive.	
Section C: Deals with environmental exposures where a person may be exposed to asbestos in situations including living and working near asbestos sources	25 experts accepted section as it is (∼83%). 5 suggested revisions (∼17%). None rejected. Some additional environmental exposures were added including asbestos textile unit.	
Section D: Deals with non-occupational sources of asbestos exposure	23 experts accepted section as it is (∼77%). 7 suggested revisions (∼23%). None rejected. Questions pertaining to duration of exposure and use of respirators was added.	
Section E: Deals with the para-occupational sources of asbestos exposure	24 experts accepted section as it is (80%). 6 suggested revisions (20%). None rejected. Professions as in Section B increased. A specific question pertaining to cleaning/washing clothes of family member added.	
Section F: Deals with other exposures (including dust exposures) which may be useful to ruling out asbestos exposure	22 experts accepted section as it is (∼73%). 8 suggested revisions (∼27%). None rejected. The duration of exposure parameter was added.	
Comments by experts on overall questionnaire (as a whole); In case there is a need for addition of a new question, or any revision.	Comments ranged from including exposure time, adding more exposure scenarios, adding anti-tubercular treatment, past CT scan, fluid drawn from chest in history. History of smoking to be included; Family cancer history to be added; Farming exposure to be added.	

## DISCUSSION

Many countries around the world, including developing countries like India should better regulate or, better yet, stop the use of asbestos and other carcinogens. This is important as preventable asbestos related diseases are likely to increase multifold.^15^ Awareness about non-occupational exposure and para-occupational exposure, along with occupational exposure to asbestos is essential as these may explain about 20% of mesotheliomas in some developed countries.^16^

The recordkeeping of mesothelioma must be prudent, as in some countries which have developed dedicated mesothelioma registries.^17^ Some steps to strengthen recordkeeping include making cancer nationally notifiable and enhancing registry programs.^18,19^ Occupational registries, if not developed, must be developed, and strengthened.^20^ Mesothelioma, in India, which was notifiable under the Mines Act, 1952 has not been included in the new Occupational Safety, Health and Working Conditions Code of 2020.^21–23^ Mesothelioma as a separate entry should be added to the list of notifiable diseases and occupational disease registries must be made operational and active. There is a further need to strengthen the general cancer registry as there may be an overlap due in reporting to occupational regulators and patients being reported in the general hospital-based cancer registries. Further, cancer as a disease needs to be made notifiable so that it becomes a legal mandate for all medical practitioners and hospitals to report cancer cases, which will include mesothelioma, to a common country wide database. This will help direct policy towards cancer hotspots so that prevention in those areas can be strengthened rather than strengthening only the cure.

There are some limitations in this work which includes, first, that experts could have been more globally diverse. Second, there is potential of bias in the selection of the experts. Third, Delphi consensus in some contexts may require more rounds. Fourth, the tool has not been field-tested for reliability and validity beyond the content consensus, and further data may be needed to validate the same clinically. And fifth, the list of sources of exposure, even though not in scope of this work, may need to be populated by a systematic review of literature and may have regional variations. Some sources of asbestos exposure leading to a possibility of mesothelioma occurrence are cited in Part B of the Supplementary Material. Such a list is not truly exhaustive as that exercise has been done in detail by other studies, and the list may increasingly expand.^24,25^ Other causes may also be speculated for mesothelioma apart from asbestos like ionizing radiation, or genetic causes, etc.^26,27^

## CONCLUSION

A questionnaire has been developed that includes occupational, non-occupational and para occupational exposure to asbestos as a risk factor for mesothelioma. Future work should include pilot implementation in clinical settings, its use in epidemiological and legal settings, and translation into local languages for greater adoption.

## Supplementary Material


