# Supplementary material for: Development of a history-taking form for mesothelioma patients at risk of exposure to asbestos
Source: Public Health Action. 2025 Dec 3;15(4):160–3. doi: 10.5588/pha.25.0024 (PMC12687119; doi:10.5588/pha.25.0024)
Supplement: Supplementary file 1 [file pha25-0024_supplementarydata1.pdf]

Supplementary Material

(Part A)

The Model History-taking Form for Clinicians Dealing with Mesothelioma Patients Validated by Delphi Expert Consensus

Note: The clinician attending the patient may fill the following history-taking form. First, the patients' details should be taken. This should be followed by the sets of questions from A to F. All questions may be asked while a detailed history is being taken. A vernacular version (provided separately) may be used if needed. The form is of 5 pages (Pages 3 to 7).

Personal Details: *Name Expunged/ Address Expunged/ Phone Number Expunged*
Age: _________
Gender: _________
Pin Code/Zip Code/Postal Code of residential address (permanent): _______
Whether Working or Retired:___________
If Retired, age of retirement: __________
If Retired, reason for retirement: _________
Started work at what age: ___________
Occupation (Current): ________
Occupation (Major period of career): _________________
Pin Code/Zip Code/Postal Code of Current workplace: _______
Other Occupations during lifetime with duration of work; pin/zip/postal code; beginning and end year:
____
____
____

A. Additional Details:

1. Past History of COPD? Yes/No? _______
2. Past History of Any other lung disease: ILD/Restrictive Disease: Asbestosis/Silicosis/Any other chest/lung disease: Yes/No? ________
3. Past History of Asthma: Yes/No?_________
4. Past History of Tuberculosis: Yes/No? _________
5. Past History of Cancer (Self): Yes/No? _________
6. Family History of Cancer: Yes/No____ Details________

If yes, please provide details: ________

7. Cough History:
   i. If one time: Yes/No? _________Details: _________
   ii. If >3 weeks: Yes/No? ________Details: ________
8. Breathlessness: Yes/No? _______
9. Weight Loss (in last one year): Yes/No? ______
10. Chest Pain: Yes/No? _______
11. Cough with blood-stained sputum: Yes/No? Details_________
12. History of Smoking/Tobacco Use: Yes/No? ___________
    i. If smoker, age of initiation:_____

ii. If smoker, Whether currently smoking: Yes/No:____ Detail:_____
iii. If quit smoking, age at which done:

13. Was any fluid drawn (fluid aspiration) ever from your chest?________
14. Any prior Anti-Tubercular Treatment (DOTS) taken: Yes/No?
    If Yes, How many months?:_____
15. Have you ever had Chest X-Ray before?: Yes/No?:_______Details:_______
a. Have you ever had Chest CT Scan before? Yes/No?:_______Details:_______
16. Any past history of pleural effusion?_________
    i. If yes, please provide details: _________

Any other remarks: __________

B. Have YOU ever worked in:

1. An Asbestos Factory: Yes/No
2. Asbestos Mine: Yes/No
3. Soapstone (Talc/Sheatite) Mine: Yes/No
4. Soapstone (Talc/Sheatite) Mill/Crushing Unit: Yes/No
5. Dolomite Mine: Yes/No
6. Dolomite Mill/Crushing Unit: Yes/No
7. Granite Mine: Yes/No
8. Granite Mill: Yes/No
9. Car/Scooter Mechanic Work: Yes/No
10. Marble Mine: Yes/No
11. Marble Cutting Unit: Yes/No
12. Talcum Powder Factory: Yes/No
13. Construction work dumping site: Yes/No
14. Construction work installing asbestos cement sheets: Yes/No
15. Construction work working with cement pipes: Yes/No
16. Construction work involved with demolition: Yes/No
17. Asbestos/Soapstone/Dolomite/Marble/Granite store/shop/trading: Yes/No
18. Shipyard: Yes/No
19. Ship Recycling Work: Yes/No
20. A Power Station: Yes/No
21. As an Electrician: Yes/No
22. As a Plumber: Yes/No
23. As a Road construction worker dealing with asphalt/tar and related machinery: Yes/No
24. A Coal mine: Yes/No
25. A diamond mine: Yes/No
26. A Circus: Yes/No
27. Fireproofing Equipment manufacturing unit: Yes/No
28. Fire Services: Yes/No
29. As a Professional Tabla Player/percussionist using talc: Yes/No
30. As a Professional or hobby carrom board player: Yes/No
31. As a Sculptor using marble/soapstone: Yes/No
32. As a person involved in gold, silver, diamond, and gemstone jewellery making industry: Yes/No
33. Laboratory work, instructor/handler where asbestos handling was performed: Yes/No

34. Asbestos Textile Factory/Workshop/Tailoring unit: Yes/No
35. Service in the Navy/Merchant Navy: Yes/No
36. Worked in an automobile brake/clutch factory: Yes/No
37. Subway/Tunnel Work: Yes/No
38. Agriculture work involving paddy/sugarcane: Yes/No
39. Any work that involves use of talcum powder: Yes/No
40. Furnace/Boiler Manufacture, Install and Repair: Yes/No
41. Construction-Marble and Tile cutting and installation: Yes/No
42. Train Cabin and Engine Manufacture/Maintenance: Yes/No
43. Stone Crusher Unit: Yes/No
44. As a waste picker/waste handler/waste processing plant or unit: Yes/No
45. As a person exposed to carbon nanotubes? Yes/No
46. Any other Mine: Yes/No (If yes, which one? - Answer below)
47. Other Work involving asbestos: Yes/No; Describe below.

If answer was Yes, please describe the work_______________________________
What was the duration of work/exposure: ___________
Remarks: _________________________

C. Have you ever lived or worked near:

a. An Asbestos Factory: Yes/No
b. Asbestos Mine: Yes/No
c. Soapstone (Talc/Sheatite) Mine: Yes/No
d. Soapstone (Talc/Sheatite) Mill/Crushing Unit: Yes/No
e. Dolomite Mine: Yes/No
f. Dolomite Mill: Yes/No
g. Granite Mine: Yes/No
h. Granite Mill: Yes/No
i. Car/Scooter Mechanic Work: Yes/No
j. Marble Mine: Yes/No
k. Marble Cutting Unit: Yes/No
l. Talcum Powder Factory: Yes/No
m. An asbestos waste dump yard: yes/no
n. A construction waste dumping site: yes/no
o. Asbestos Cement Sheet Factory: Yes/No
p. Cement Pipe Factory: Yes/No
q. Asbestos/Soapstone/Dolomite/Marble/Granite store/shop/trading: Yes/No
r. Ship Recycling Unit: Yes/No
s. Any construction site where marble/granite/tile cutting was taking place: Yes/No
t. Brake/clutch manufacturing unit: Yes/No
u. Asbestos textile unit: Yes/No
v. Any other place where asbestos/talcum/marble related work was performed: Yes/No

If answer was Yes, please describe _______________________________

D. Have you ever used:

a. An asbestos sheet roofing: Yes/No

b. Asbestos insulation: Yes/No
c. Loose or sprayed asbestos for insulation: Yes/No
d. Talcum Powder for personal use: Yes/No. if Yes, Details: ______________
e. Talcum powder as part of your professional work: Yes/No
f. Asbestos Cement Pipe: Yes/No
g. Asbestos textile like asbestos gloves etc: Yes/No

Remarks: If Any answer is Yes, what was the duration of exposure; Any other details: __________

h. Have you ever used a respirator or personal protective equipment during your occupation? Yes/No: _____
Details: _______

E. Has your MOTHER/FATHER/FAMILY MEMBER/OTHER PERSON LIVING WITH YOU ever worked in:

1. An Asbestos Factory: Yes/No
2. Asbestos Mine: Yes/No
3. Soapstone (Talc/Sheatite) Mine: Yes/No
4. Soapstone (Talc/Sheatite) Mill/Crushing Unit: Yes/No
5. Dolomite Mine: Yes/No
6. Dolomite Mill/Crushing Unit: Yes/No
7. Granite Mine: Yes/No
8. Granite Mill: Yes/No
9. Car/Scooter Mechanic Work: Yes/No
10. Marble Mine: Yes/No
11. Marble Cutting Unit: Yes/No
12. Talcum Powder Factory: Yes/No
13. Construction work dumping site: Yes/No
14. Construction work installing asbestos cement sheets: Yes/No
15. Construction work working with cement pipes: Yes/No
16. Construction work involved with demolition: Yes/No
17. Asbestos/Soapstone/Dolomite/Marble/Granite store/shop/trading: Yes/No
18. Shipyard: Yes/No
19. Ship Recycling Work: Yes/No
20. A Power Station: Yes/No
21. As an Electrician: Yes/No
22. As a Plumber: Yes/No
23. As a Road construction worker dealing with asphalt/tar and related machinery: Yes/No
24. A Coal mine: Yes/No
25. A diamond mine: Yes/No
26. A Circus: Yes/No
27. Fireproofing Equipment manufacturing unit: Yes/No
28. Fire Services: Yes/No
29. As a Professional Tabla Player: Yes/No
30. As a Professional or hobby carrom board player: Yes/No
31. As a Sculptor using marble/soapstone: Yes/No
32. As a person involved in gold, silver, diamond, and gemstone jewellery making industry: Yes/No

33. Laboratory work, instructor/handler where asbestos handling was performed: Yes/No
34. Asbestos Textile Factory/Workshop/Tailoring unit: Yes/No
35. Service in the Navy: Yes/No
36. Worked in an automobile brake/clutch factory: Yes/No
37. Subway/Tunnel Work: Yes/No
38. Agriculture work involving paddy/sugarcane: Yes/No
39. Any work that involves use of talcum powder: Yes/No
40. Furnace/Boiler Manufacture, Install and Repair: Yes/No
41. Construction-Marble and Tile cutting and installation: Yes/No
42. Train Cabin and Engine Manufacture/Maintenance: Yes/No
43. Stone Crusher Unit: Yes/No
44. As a waste picker/waste handler/waste processing plant or unit: Yes/No
45. As a person exposed to carbon nanotubes? Yes/No
46. Any other Mine: Yes/No (If yes, which one? - Answer below)
47. Other Work involving asbestos: Yes/No; Describe below.

If answer was Yes, please describe the work______________________________
Relationship with family member exposed: ______________________________
Remarks: ________________________
Also, please provide the duration of exposure to above: _______

48. Has any of your MOTHER/FATHER/FAMILY MEMBER LIVING WITH YOU used talcum powder? Yes/No_____ Describe:_________
49. Have you been involved with the cleaning, washing, ironing of clothes of your MOTHER/FATHER/FAMILY MEMBER LIVING WITH YOU? Yes/No_______ Describe:__________

F. Any other exposure to any other dust? Yes/No?________
If Yes, which type of dust?:_________
If yes, was it metallic/chemical or mineral dust, Describe: ________
What was the duration of exposure? _________

Any other Remarks:

____
____

(Part B)

Table: A non-exhaustive list of some sources of asbestos exposure from literature and possibility of mesothelioma occurrence.

S. No.	Occupation	Citation
1.	List of all occupations from the US national mesothelioma virtual bank.	Gao, et. al, 2023[1]
2.	Others including welders, flame cutters, boiler makers, structural metalworkers, etc.	DeBono, et. al, 2021[2]
3.	Construction workers from Italian Mesothelioma Register	Vimercati, et. al, 2023[3]
4.	Chrysotile mine workers	Schuz, et. al, 2024[4]
5.	Asbestos-Cement Factory workers	Ulvestad et. al., 2002, Jadhav, et, al, 2012 [5,6]
6.	School teachers	Lilienfield, 1991[7]
7.	Navy personnel	Lemen & Landrigan, 2021[8]
8.	Diamond mine workers	Nelson et. al, 2011[9]
9.	Coal mine (Related to associated minerals, or equipment and not coal itself)	Gothi, et. al, 2015[10]
10.	Jewellers	Kern, et. al, 1992[11]
11.	Circus workers (Attributed to equipment or props used)	Mensi, et. al, 2021[12]
12.	Automotive dealerships	Frank, 2023[13]
13.	Friction material, clutch manufacturing	Gothi, et. al, 2016[14]
14.	Railroad machinists	Mancuso, 1988[15]
15.	Commercial talc	Gordon, et. al, 2014, Fitzgerald, et. al, 2019, Moline, et al, 2020 and 2023.[16–19]
16.	Neighbourhood exposure	Kitamura, et. al, 2023[20]
17.	Wives of asbestos workers	Ferrante, et. al, 2007[21]
18.	Mine tailings from an abandoned mines	Jadhav, et. al, 2025[22]
19.	Oil Industry	Mclaren Berge, et. al, 2024[23]
20.	Power Plants	Crosignani, et. al, 1995[24]
21.	Shipbuilding	Hemminki, et. al, 2021, Singh, et. al, 2020[25,26]
22.	Marble work/disposal	Singh, et. al, 2025[27]
23.	Use and disposal of asbestos-cement and allied material roofing.	Obminski, 2022, Kang, et el, 2018, Oberta, et, al, 2018 [28–31]

References:

1. Gao Y, Mazurek JM, Li Y, et al. Industry, occupation, and exposure history of mesothelioma patients in the U.S. National Mesothelioma Virtual Bank, 2006–2022. *Environmental Research*. 2023;230:115085. doi:10.1016/j.envres.2022.115085
2. DeBono NL, Warden H, Logar-Henderson C, et al. Incidence of mesothelioma and asbestosis by occupation in a diverse workforce. *American J Industrial Med*. 2021;64(6):476-487. doi:10.1002/ajim.23245

3. Vimercati L, Cavone D, De Maria L, et al. Mesothelioma Risk among Construction Workers According to Job Title: Data from the Italian Mesothelioma Register. *La Medicina del Lavoro*. 2023;114(3):e2023025. doi:10.23749/mdl.v114i3.14538
4. Schüz J, Kovalevskiy E, Olsson A, et al. Cancer mortality in chrysotile miners and millers, Russian Federation: main results (Asbest Chrysotile Cohort-Study). *JNCI: Journal of the National Cancer Institute*. 2024;116(6):866-875. doi:10.1093/jnci/djad262
5. Jadhav A, Roy N. Asbestosis: Past voices from the Mumbai factory floor. *Indian J Occup Environ Med*. 2012;16(3):131. doi:10.4103/0019-5278.111758
6. Ulvestad B, Kjærheim K, Martinsen JI, et al. Cancer incidence among workers in the asbestos-cement producing industry in Norway. *Scand J Work Environ Health*. 2002;28(6):411-417. doi:10.5271/sjweh.693
7. Lilienfeld DE. Asbestos-Associated Pleural Mesothelioma in School Teachers: A Discussion of Four Cases[a]. *Annals of the New York Academy of Sciences*. 1991;643(1):454-458. doi:10.1111/j.1749-6632.1991.tb24494.x
8. Lemen RA, Landrigan PJ. Sailors and the Risk of Asbestos-Related Cancer. *IJERPH*. 2021;18(16):8417. doi:10.3390/ijerph18168417
9. Nelson G, Murray J, Phillips JI. The Risk of Asbestos Exposure in South African Diamond Mine Workers. *The Annals of Occupational Hygiene*. Published online July 2011. doi:10.1093/annhyg/mer028
10. Gothi D, Verma A, Sah R. An unusual case of mesothelioma. *Lung India*. 2015;32(2):194. doi:10.4103/0970-2113.152665
11. Kern DG, Hanley KT, Roggli VL. Malignant mesothelioma in the jewelry industry. *American J Industrial Med*. 1992;21(3):409-416. doi:10.1002/ajim.4700210313
12. Mensi C, Zellino C, Polonioli M, et al. Pleural mesothelioma in a circus worker. *Journal of Occupational Health*. 2021;63(1):e12250. doi:10.1002/1348-9585.12250
13. Frank AL. Four mesothelioma cases from a single automotive dealership: A case series. *American J Industrial Med*. 2023;66(10):904-906. doi:10.1002/ajim.23521
14. Gothi D, Gahlot T, Sah R, et al. Asbestos-induced lung disease in small-scale clutch manufacturing workers. *Indian J Occup Environ Med*. 2016;20(2):95. doi:10.4103/0019-5278.197533
15. Mancuso TF. Relative risk of mesothelioma among railroad machinists exposed to chrysotile. *American J Industrial Med*. 1988;13(6):639-657. doi:10.1002/ajim.4700130604
16. Moline J, Bevilacqua K, Alexandri M, Gordon RE. Mesothelioma Associated With the Use of Cosmetic Talc. *Journal of Occupational & Environmental Medicine*. 2020;62(1):11-17. doi:10.1097/JOM.0000000000001723
17. Moline J, Patel K, Frank AL. Exposure to cosmetic talc and mesothelioma. *J Occup Med Toxicol*. 2023;18(1):1. doi:10.1186/s12995-023-00367-5
18. Fitzgerald S, Harty E, Joshi TK, Frank AL. Asbestos in commercial Indian talc. *American J Industrial Med*. 2019;62(5):385-392. doi:10.1002/ajim.22969
19. Gordon RE, Fitzgerald S, Millette J. Asbestos in commercial cosmetic talcum powder as a cause of mesothelioma in women. *International Journal of Occupational and Environmental Health*. 2014;20(4):318-332. doi:10.1179/2049396714Y.0000000081
20. Kitamura Y, Zha L, Liu R, et al. Association of mesothelioma deaths with neighborhood asbestos exposure due to a large-scale asbestos-cement plant. *Cancer Science*. 2023;114(7):2973-2985. doi:10.1111/cas.15802

21. Ferrante D, Bertolotti M, Todesco A, Mirabelli D, Terracini B, Magnani C. Cancer Mortality and Incidence of Mesothelioma in a Cohort of Wives of Asbestos Workers in Casale Monferrato, Italy. *Environ Health Perspect*. 2007;115(10):1401-1405. doi:10.1289/ehp.10195
22. Jadhav AV, Gawde N, Veerappan R, Choi Y, Frank AL. Understanding exposure risk using soil testing and GIS around an abandoned asbestos mine. *Annals of Global Health*. 2025;91(1):2. doi:10.5334/aogh.4624
23. Berge LAM, Shala NK, Barone-Adesi F, et al. Exposure to fibres and risk of pleural mesothelioma in the Norwegian Offshore Petroleum Workers cohort. *Occup Environ Med*. 2024;81(7):331-338. doi:10.1136/oemed-2024-109424
24. Crosignani P, Forastiere F, Petrelli G, et al. Malignant mesothelioma in thermoelectric power plant workers in Italy. *American J Industrial Med*. 1995;27(4):573-576. doi:10.1002/ajim.4700270410
25. Singh R, Cherrie JW, Rao B, Asolekar SR. Assessment of the future mesothelioma disease burden from past exposure to asbestos in ship recycling yards in India. *International Journal of Hygiene and Environmental Health*. 2020;225:113478. doi:10.1016/j.ijheh.2020.113478
26. Hemminki K, Försti A, Chen T, Hemminki A. Incidence, mortality and survival in malignant pleural mesothelioma before and after asbestos in Denmark, Finland, Norway and Sweden. *BMC Cancer*. 2021;21(1):1189. doi:10.1186/s12885-021-08913-2
27. Singh R, Fitzgerald S, Dada R, Frank AL. Marble Waste Dump Yard in Rajasthan, India Revealed as a Potential Asbestos Exposure Hazard. *IJERPH*. 2025;22(2):215. doi:10.3390/ijerph22020215
28. Oberta AF, Poye L, Compton SP. Releasability of asbestos fibers from weathered roof cement. *Journal of Occupational and Environmental Hygiene*. 2018;15(6):466-473. doi:10.1080/15459624.2018.1448401
29. Obmiński A. Asbestos cement products and their impact on soil contamination in relation to various sources of anthropogenic and natural asbestos pollution. *Science of The Total Environment*. 2022;848:157275. doi:10.1016/j.scitotenv.2022.157275
30. Kang D, Kim YY, Shin M, et al. Relationships of Lower Lung Fibrosis, Pleural Disease, and Lung Mass with Occupational, Household, Neighborhood, and Slate Roof-Dense Area Residential Asbestos Exposure. *IJERPH*. 2018;15(8):1638. doi:10.3390/ijerph15081638
31. Kottek M, Yuen ML. Public health risks from asbestos cement roofing. *American J Industrial Med*. 2022;65(3):157-161. doi:10.1002/ajim.23321
